# Electric Pulse Stimulation of Myotubes as an *In Vitro* Exercise Model: Cell-Mediated and Non-Cell-Mediated Effects

**DOI:** 10.1038/srep10944

**Published:** 2015-06-19

**Authors:** Inkie J.A. Evers-van Gogh, Sheril Alex, Rinke Stienstra, Arjan B. Brenkman, Sander Kersten, Eric Kalkhoven

**Affiliations:** 1 Molecular Cancer Research and Center for Molecular Medicine, University Medical Centre Utrecht, Universiteitsweg 100, 3584 CG, Utrecht, The Netherlands; 2 Nutrition, Metabolism and Genomics Group, Division of Human Nutrition, Wageningen University, PO BOX 8129, 6700 EV Wageningen, the Netherlands; 3 Department of Medicine, Radboud University Medical Centre, Geert Grooteplein 8, P.O. Box 9101, Nijmegen 6500 HB, The Netherlands

## Abstract

Regular exercise has emerged as one of the best therapeutic strategies to prevent and treat type-2-diabetes. Exercise-induced changes in the muscle secretome, consisting of myokines and metabolites, may underlie the inter-organ communication between muscle and other organs. To investigate this crosstalk, we developed an *in vitro* system in which mouse C2C12 myotubes underwent electric pulse stimulation (EPS) to induce contraction. Subsequently the effects of EPS-conditioned media (EPS-CM) on hepatocytes were investigated. Here, we demonstrate that EPS-CM induces *Metallothionein 1/2* and *Slc30a2* gene expression and reduces *Cyp2a3* gene expression in rat hepatocytes. When testing EPS-CM that was generated in the absence of C2C12 myotubes (non-cell EPS-CM) no decrease in Cyp2a3 expression was detected. However, similar inductions in hepatic *Mt1/2* and *Slc30a2* expression were observed. Non-cell EPS-CM were also applied to C2C12 myotubes and compared to C2C12 myotubes that underwent EPS: here changes in AMPK phosphorylation and myokine secretion largely depended on EPS-induced contraction. Taken together, these findings indicate that EPS can alter C2C12 myotube function and thereby affect gene expression in cells subjected to EPS-CM (Cyp2a3). However, EPS can also generate non-cell-mediated changes in cell culture media, which can affect gene expression in cells subjected to EPS-CM too. While EPS clearly represents a valuable tool in exercise research, care should be taken in experimental design to control for non-cell-mediated effects.

Over the past few decades, the prevalence of obesity and its associated complications, type 2 diabetes and cardiovascular disease, have increased to a great extent^1^,^2^. Regular exercise has emerged as one of the best therapeutic strategies to prevent and treat these diseases, often partially independent of weight loss^3^,^4^,^5^. While muscle is the primary organ affected by exercise, beneficial effects can be observed on a variety of distant organ systems, such as the brain, heart, lungs, adipose tissue and liver^6^. The means by which exercise mediates these beneficial effects on distant target organs are largely undefined.

Recently, muscle has been identified as an active secretory organ. The cytokines and peptides released from the muscle, exerting autocrine, paracrine and endocrine functions, are classified as myokines (reviewed in^7^). New myokines are constantly being identified, using different *in vivo* (mouse and human) and *in vitro* (cellular) models. For example, muscle-specific overexpression of PGC1α, a transcriptional coactivator that is induced by exercise and drives several of the beneficial effects of exercise in muscle, resulted in the identification of Irisin^8^. Irisin is an exercise-induced myokine that has been suggested to primarily target white adipose tissue, resulting in conversion to a brown-like phenotype and increased thermogenesis^8^. Muscle-specific overexpression of a specific PGC1α isoform (PGC1-α4) in mice led to the identification of the myokine meteorin-like (Metrnl), which was shown to reduce adipose tissue inflammation and thereby stimulates thermogenesis^9^. In an alternative approach, we recently identified and validated chemokine (C-X3-C motif) ligand 1 (CX3CL1) and Monocyte Chemoattractant Protein (MCP-1) as exercise-induced myokines in humans by microarray-based analysis of secreted proteins in a one-legged acute endurance exercise study^10^,^11^. Cultured myotubes (mouse or human) have also been used in several studies to identify myokines, often subjected to electric pulse stimulation (EPS) to induce contraction and thereby mimic exercise^12^,^13^. Such approaches have for example resulted in the identification of Leukemia Inhibitory Factor (LIF)^14^, pigment epithelium derived factor (PEDF) and dipeptidyl peptidase 4 (DPP4)^12^ as exercise-induced myokines. Besides myokines, skeletal muscle also secretes metabolites into the circulation, as revealed by metabolomic approaches^15^,^16^. At least one of these metabolites, β-aminoisobutyric acid (BAIBA), may function in an endocrine fashion, as it has been suggested to induce browning of white adipose tissue and increase β-oxidation in the liver^16^.

While these recent studies provide proof-of-principle that myokines and/or muscle-derived metabolites can mediate the crosstalk between exercising muscle and distant target organs (especially white adipose tissue^8^,^9^,^16^), effects on the liver were not always investigated. In addition, as the muscle secretome consists of hundreds of myokines and metabolites^11^,^12^,^16^,^17^,^18^,^19^,^20^,^21^,^22^, muscle-liver communication may depend on the combination of several (un)identified factors present in the exercise-induced secretome. In the present study we therefore subjected C2C12 myotubes to EPS, collected the contraction induced secretome (EPS conditioned media; EPS-CM), and investigated the effects of this EPS-CM on hepatic gene expression in FAO hepatocytes and primary hepatocytes. Here, we demonstrate that EPS-CM induces *Metallothionein 1/2* and *Slc30a2* gene expression and reduces *Cyp2a3* gene expression in rat hepatocytes. However, EPS-CM generated in the absence of C2C12 myotubes also induced *Metallothionein 1/2* and *Slc30a2* gene expression, indicating that EPS can induce both cell-mediated (*Cyp2a3*) and non-cell-mediated effects. Direct effects of EPS on C2C12 myotube function (AMPK phosphorylation, myokine secretion) were mainly caused by EPS-induced contraction and not due to EPS-induced changes in cell culture media. Taken together, these findings indicate that EPS clearly represents a valuable tool in exercise research, but care should be taken in experimental design to control for non-cell-mediated effects.

## Methods

### Cell Culture

Mouse skeletal muscle C2C12 cells were maintained in DMEM containing 4.5 g/l glucose (Lonza, Basel, Switzerland) supplemented with 10% heat inactivated Fetal Bovine Serum (FBS) (Gibco/Life Technologies, Carlsbad, CA, USA), 100 μg/ml penicillin and 100 μg/ml streptomycin (Lonza) (growth medium). Differentiation was induced by switching from growth medium to DMEM containing 4.5 g/l glucose supplemented with 2% Horse Serum (Gibco/Life Technologies), 100 μg/ml penicillin and 100 μg/ml streptomycin (differentiation medium) and the differentiation medium was changed every day. Differentiation was started when cells reached ~80–90% confluence (day 0). Rat hepatoma FAO cells were maintained in DMEM containing 4.5 g/l glucose supplemented with 10% FBS, 100 μg/ml penicillin and 100 μg/ml streptomycin. Primary hepatocytes were isolated from Wistar-HsdCpb:WU rats (Harlan, Horst, The Netherlands) by two-step collagenase perfusion^23^. Cells were plated on collagen-coated plates at a density of 9 × 10^4^ viable cells/cm^2^ and cultured in DMEM containing 4.5 g/l glucose supplemented with 10% heat inactivated FBS, 100 μg/ml penicillin and 100 μg/ml streptomycin. Treatment was started and fresh medium was added 3–4 hours after plating.

### Electric pulse stimulation

Electric pulse stimulation experiments were performed according to Lambernd *et al.*^24^. In short, myotubes were differentiated until day 5, followed by overnight starvation in DMEM without FBS (starvation medium) to exclude effects of the many undefined factors in FBS. Starvation medium was refreshed directly before stimulation to minimize effects of factors potentially secreted during the overnight starvation. EPS, with the conditions of 1 Hz frequency, 2 ms pulse duration and 11.5 V intensity, was applied using a C-dish with carbon electrodes combined with a pulse generator for 24 hours (C-Pace 100; IonOptix, Milton, MA, USA). Conditioned media (CM) with and without EPS were collected, pooled, centrifuged at 800 rpm for 5 min and stored at −80 °C. A 1:1 ratio of growth medium (10% FBS) and CM (0% FBS) was added to FAO hepatocytes and primary hepatocytes for the indicated time points. Myotubes were used for RNA isolation or protein isolation (see RNA isolation and Real-time PCR and Western blotting).

### Conditioned Medium Fractionation

Control-CM and EPS-CM was fractionated into a protein fraction and a small molecule fraction using Vivaspin 2 columns with 5,000 MWCO (Hydrosart®) Membrane (Generon, Berkshire, UK). Methanol:chloroform extraction was used to fractionate the small molecule/metabolite fraction into polar and non-polar molecules. In short, A 1:1:1 ratio of CM, methanol and chloroform was vortexed for 2 minutes, rotated for 10 minutes and centrifuged at 14.000 rpm for 10 min at 4 °C. The polar/methanol fraction and apolar/chloroform fraction were dried, dissolved in DMEM containing 4.5 g/l glucose supplemented with 100 μg/ml penicillin and 100 μg/ml streptomycin and added to FAO hepatocytes for the indicated time points.

### ELISA

CM were analyzed using ELISA to measure the secretion of IL-6, MCP-1 and KC. DuoSet ELISA Development kits against mouse IL-6, MCP-1 and KC were used (R&D Systems, Minneapolis, MN, USA). The cytokine concentrations were quantified by using a mouse recombinant IL-6, MCP-1 and KC as standard. The measurements were performed exactly following the manufacturers’ protocols. For statistical analysis Student t-tests were used.

### Affymetrix microarray

Microarray analysis was performed on RNA of FAO hepatocytes stimulated with C2C12 CM for 2 and 16 hours. The RNA of 4 individual experiments was extracted using TRIzol reagent (Invitrogen, Carlsbad, CA, USA), purified using the RNeasy Micro Kit (Qiagen, Germantown, MD, USA) and the integrity were verified with the RNA 6000 Nano assay on the Agilent 2100 Bioanalyzer (Agilent Technologies, Amsterdam, the Netherlands). Hybridization, washing, and scanning of the Affymetrix GeneChip Rat Gene 1.0 ST Array were performed on Affymetrix GeneTitan. Scans of the Affymetrix arrays were processed using packages from the Bioconductor project^25^. Raw signal intensities were obtained by robust multiarray (RMA) normalization^26^.

### RNA isolation and Real-time PCR

Total RNA from cultured cells and mice livers was extracted using TRIzol reagent (Invitrogen). Reverse transcription was performed using Superscript II and oligo(dT) primers (Invitrogen). PCR-amplifications were carried out using iQ SYBR Green Supermix on a MyIQ real time PCR detection system (Bio-Rad, Hercules, CA, USA).

The sequences of the primers used for real-time PCR are as follows: rMt1 forward, GCT GTG TCT GCA AAG GTG C; rMt1 reverse, ATT TAC ACC TGA GGG CAG CA; rMt2 forward, AAG AAA AGC TGC TGT TCC TGC; rMt2 reverse, CTG CAC TTG TCC GAA GCC T; rSlc30a2 forward, CCA GTG TCC GAG CTG CCT T; rSlc30a2 reverse, GAT GGA GAA GAG GAA GGT GC; rCyp2a3 forward, TGT CTG TCT GGA AGC AGA GG; rCyp2a3 reverse, GGA TGG TGA ATA CAG GAC CG; r36B4 forward, CGG GAA GGC TGT GGT GCT GAT G; r36B4 reverse, TCG GTG AGG TCC TCC TTG GTG AAC; rβ-Actin forward, CTG GCT CCT AGC ACC ATG A; rβ-Actin reverse, TAG AGC CAC CAA TCC ACA CA; mIl-6 forward, CAT CCA GTT GCC TTC TTG GG; mIl-6 reverse, CCA GTT TGG TAG CAT CCA TC; mMcp-1 CTT CTG GGC CTG CTG TTC A; mMcp-1 reverse, CCA GCC TAC TCA TTG GGA TCA; mKc forward, ACT GCA CCC AAA CCG AAG TC; mKc reverse, TGG GGA CAC CTT TTA GCA TCT T; mTbp forward, GGG GAG CTG TGA TGT GAA GT; mTbp reverse, CCA GGA AAT AAT TCT GGC TCA; mβ-Actin forward, CTA AGG CCA ACC GTG AAA AG; and mβ-Actin reverse, ACT TGT CGG AAG CCT CTT TG. The rat genes were normalized to 36B4 and B-actin, while all mouse genes were normalized to TBP and B-actin. Primer efficiencies were determined using LinRegPCR v11.1^27^. Relative expression of the transcript levels was calculated as described previously^28^. For statistical analysis Student t-tests were used.

### Western blotting

To obtain total protein extracts from differentiated myotubes, cells were washed with ice-cold PBS before adding cold lysis buffer (25 mM Tris HCL pH = 7.9, 5 mM MgCl2, 10% glycerol, 100 mM KCl; 1% NP40; 0.3 mM dithiothreitol, 5 mM sodium pyrophosphate, 1 mM sodium orthovanadate, 50 mM sodium fluoride, containing freshly added protease inhibitor cocktail (Roche Applied Science, Penzberg, Germany)^29^. Cells were scraped, homogenized with a 25-gauge needle and centrifuged at 14.000 rpm for 10 min at 4 °C. Supernatants were collected and boiled with Laemmli sample buffer. Cell lysates were subjected to SDS-Page and proteins were transferred to polyvinylidene difluoride membrane (Immobilon, Millipore, Billerica, MA, USA). Membranes were blocked and incubated with anti-AMPKα, anti-phospho-AMPKα (Thr 172) (Cell Signaling Technology, Danvers, MA, USA) and anti-Actin (Sigma Aldrich). Quantification was carried out using ImageJ 1.49 m. For statistical analysis Student t-tests were used.

## Results

### EPS as an exercise mimic in C2C12 myotubes

**Figure 1:**
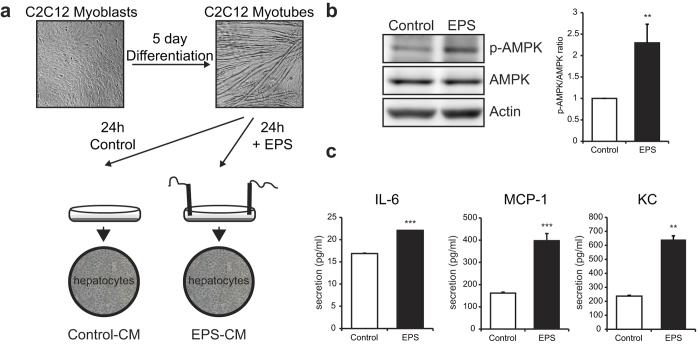
C2C12 electric pulse stimulation as a model to investigate muscle-liver crosstalk. A: Experimental set-up, stimulation of FAO hepatocytes and primary rat hepatocytes with conditioned medium of C2C12 with and without Electric Pulse Stimulation (EPS) for 24 h. B: Representative Western blot analysis of phosphorylation levels of AMPK in C2C12 cells with and without EPS (left panel). Quantification of p-AMPK over AMPK ratio was performed on 3 individual experiments (right panel). C: Concentrations of IL-6, MCP-1 and KC in the conditioned medium of C2C12 cells with and without EPS measured by ELISA. Error bars represent SD values. ** = p < 0.01; *** = p < 0.001.

In agreement with well-established protocols, after 5 days of differentiation in 2% horse serum, the majority of the C2C12 myoblasts had fused together and formed multinucleated myotubes^30^,^31^. We applied EPS (1 Hz frequency, 2 ms pulse duration and 11.5 V intensity) to these myotubes for 24 hours, which resulted in contraction of the myotubes (Fig. 1A)^24^. No other dramatic morphological changes were detected after 24 hours of EPS. Exercise and contraction increase ATP consumption in skeletal muscle and in primary skeletal muscle cultures, which causes phosphorylation and activation of AMPK^24^,^32^,^33^. To confirm that the EPS-induced contraction of C2C12 myotubes is an exercise-like condition, we examined AMPK phosphorylation and secretion of the myokines IL-6, MCP-1 and KC, the functional homolog of human IL-8. We detected a clear induction in the p-AMPK/AMPK ratio after 24 hours of EPS (Fig. 1B). In addition, the EPS-induced contraction affected the secretion of the myokines IL-6, MCP-1 and KC: EPS significantly up-regulated IL-6 secretion, reaching a concentration of 22 pg/ml after 24 hours EPS, compared with 17 pg/ml in the control situation, MCP1 was induced from 162 pg/ml to 397 pg/ml and KC from 237 pg/ml to 642 pg/ml (Fig. 1C).

### EPS-conditioned media alter gene expression in hepatocytes

**Figure 2:**
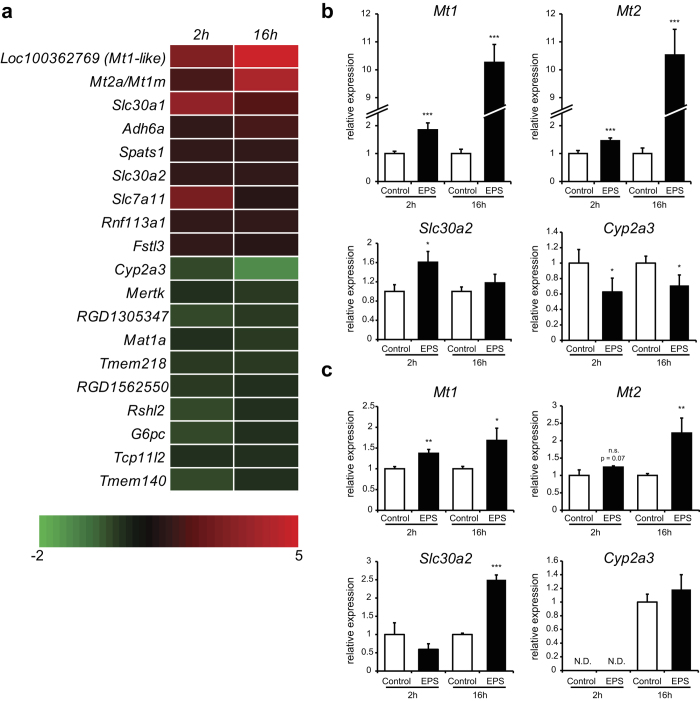
EPS conditioned medium alters gene expression in FAO hepatocytes and primary rat hepatocytes. **A**: Affymetrix microarray analysis of FAO hepatocytes stimulated with C2C12 conditioned media (CM). Four individual experiments were performed and the genes significantly (p < 0.05) up- and down-regulated (up, FC > 1.15; down, FC < −1.15) after both 2 h and 16 h stimulation are depicted. Inductions shown are compared to 2 hour and 16 hour stimulation with CM without EPS. Color scale ranges from a signal log ratio of −2 (green) to 5 (red). **B**: Relative gene expression of *Mt1, Mt2, Slc30a2* and *Cyp2a3* in FAO hepatocytes measured by RT-PCR. **C**: Relative gene expression of *Mt1, Mt2, Slc30a2* and *Cyp2a3* in primary rat hepatocytes measured by RT-PCR. (**B-C**) Error bars represent SD values of biological triplicates. * = p < 0.05; ** = p < 0.01; *** = p < 0.001 compared to CM without EPS at the same time point.

As a model to investigate the crosstalk between muscle and liver during exercise, FAO hepatocytes were incubated with a mixture of normal growth medium and C2C12 CM in a 1:1 ratio (Fig 1A). The FAO hepatocytes were incubated for 2 and 16 hours with the medium mixture to examine short-term and long-term effects on gene expression, respectively. Microarray analysis was performed on 4 biological replicates. In total, 9 genes were significantly upregulated (Fold change (FC) > 1.15; p < 0.05) and 10 genes were significantly downregulated (FC < −1.15; p < 0.05) after treatment with EPS-CM for both the 2 and 16 hour time point (Fig. 2A). We confirmed the upregulation of *Metallothionein (Mt) 1* and *2* genes, which encode small cysteine rich proteins that bind metals such as zinc and copper and acts as antioxidants^34^ and of *Slc30a2*, which encodes a zinc transporter^34^ by RT-PCR analysis (Fig 2B). RT-PCR analysis also confirmed the downregulation of *Cyp2a3 (Cyp2a6* in humans and *Cyp2a5* in mice), which is a cytochrome P450 isoform responsible for the activation of nitrosamines^35^,^36^. To investigate these gene expression changes in a more physiologically relevant *ex vivo* system, cultured primary rat hepatocytes were also incubated with control-CM and EPS-CM. The induction of *Mt1* and *Mt2* mRNA expression in hepatocytes by the contraction-induced muscle secretome was confirmed in this additional setting (Fig. 2C). The induction of *Slc30a2* was also confirmed, although in primary hepatocytes the induction was only seen after 16 hours of treatment instead of after 2 hours in FAO hepatocytes (Fig. 2C). The downregulation of *Cyp2a3* expression observed in FAO hepatocytes could not be confirmed in primary hepatocytes: expression was not detectable after 2 hours of treatment and the 16 hour time point did not show a reduction (Fig. 2C).

### Polar small molecules are predominantly responsible for the hepatic Mt1/2 induction

**Figure 3:**
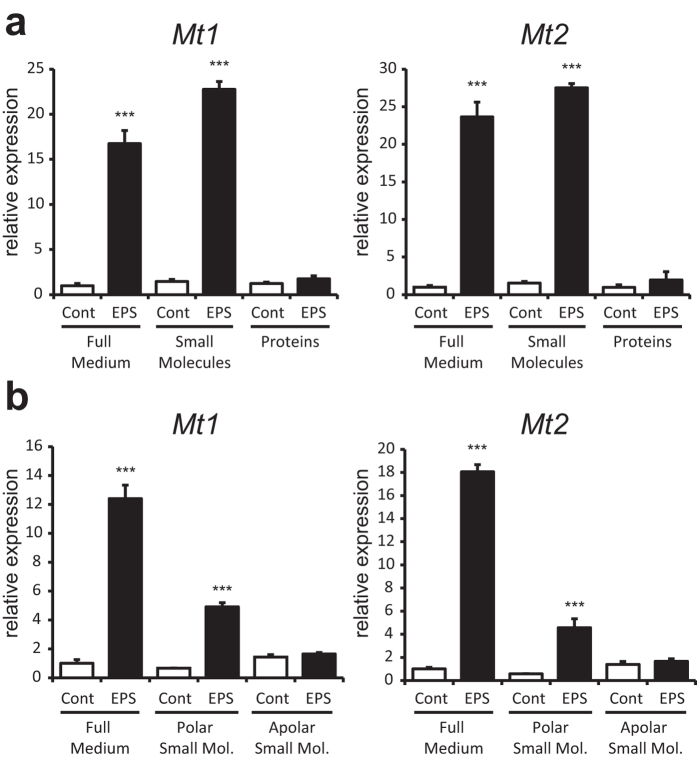
A polar small molecule regulates the induction of Metallothionein expression by C2C12 CM. Relative gene expression of *Mt1* and *Mt2* was measured by RT-PCR. **A**: FAO hepatocytes were treated with full CM, a protein fraction (>5 kDa) and a small molecule fraction (<5 kDa) for 16 hours. **B**: FAO hepatocytes were treated with full CM, a polar small molecule fraction and an apolar small molecule fraction for 16 hours. Error bars represent SD values of biological triplicates. *** = p < 0.001 compared to CM without EPS from the same fraction.

As *Mt1/2* expression was consistently induced by EPS-CM in both FAO hepatocytes and primary hepatocytes, we wished to identify the molecule(s) mediating this effect. EPS-CM was fractionated into a protein fraction and a small molecule fraction using a 5 kD cut-off column, and FAO hepatocytes were subjected to either fraction. As shown in Fig. 3A, *Mt1/2* expression was clearly induced by the small molecule fraction, while the >5 kD fraction, containing myokines and other large macromolecules, had no significant effect. Subsequent fractionation of the small molecule fraction into polar and non-polar molecules (using a methanol:chloroform extraction) indicated that polar small molecules were predominantly responsible for the *Mt1/2* induction (Fig. 3B).

### Non-cell EPS-CM can alter gene expression in hepatocytes

**Figure 4:**
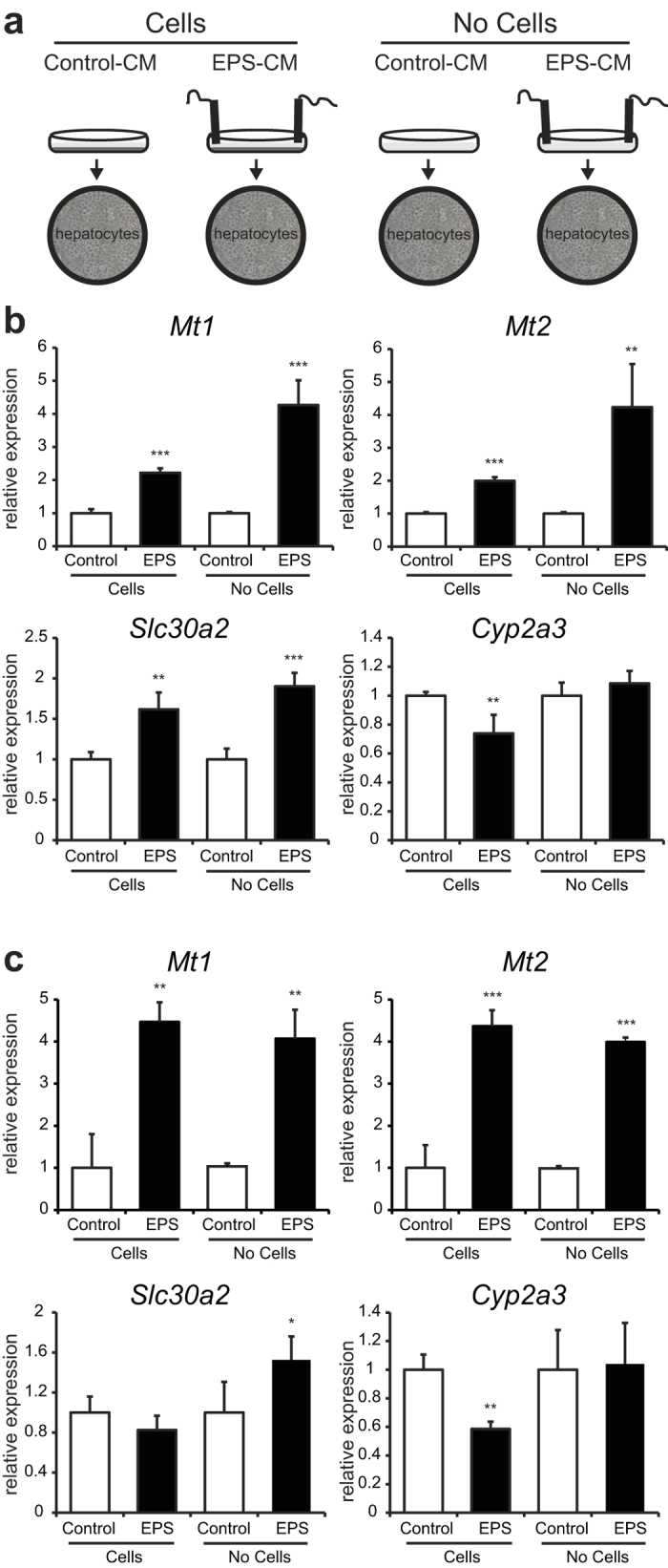
EPS can cause cell-independent changes in cell culture media that can subsequently affect hepatic gene expression. **A**: Experimental set-up, stimulation of FAO hepatocytes with EPS-CM generated with and without C2C12 cells. **B**: Relative gene expression of *Mt1, Mt2, Slc30a2* and *Cyp2a3* after 2 hour treatment was measured by RT-PCR. C: Relative gene expression of *Mt1, Mt2, Slc30a2* and *Cyp2a3* after 16 hour treatment was measured by RT-PCR. Error bars represent SD values of biological triplicates. ** = p < 0.01; *** = p < 0.001 compared to CM without EPS.

As the *in vitro* model we used to investigate the crosstalk between muscle and liver during exercise is not reported in literature thus far, we also generated EPS-CM in the absence of C2C12 myotubes as an additional control (Fig. 4A). FAO hepatocytes were subjected to this non-cell EPS-CM for 2 hours (Fig. 4B) and 16 hours (Fig. 4C) and gene expression was analyzed. For both time points a similar induction of *Mt1* and *Mt2* expression was observed in EPS-CM generated with and without C2C12 cells (Fig. 4B,C), and the same effect was observed for *Slc30a2* at the 2 hour time point (Fig. 4B). The EPS-mediated reduction in *Cyp2a3* gene expression was only observed in medium generated in the presence of C2C12 cells (Fig 4B,C). These findings suggest that next to the cell-dependent changes in *Cyp2a3* mRNA expression, EPS (and/or the electrodes) can also cause cell-independent changes in cell culture media that can subsequently affect hepatic gene expression.

### EPS causes cell-dependent changes in C2C12 myotube function

**Figure 5:**
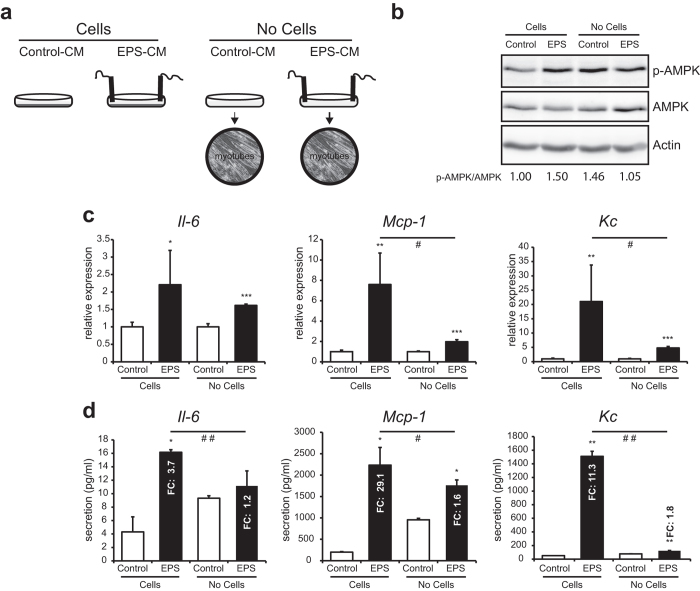
EPS causes cell-dependent changes in C2C12 myotube function. **A**: Experimental set-up, stimulation of C2C12 cells with EPS for 24 h or stimulation of C2C12 cells with EPS-CM generated without C2C12 cells for 24 h. **B**: Western blot analysis of phosphorylation levels of AMPK in C2C12 cells. Quantified phosphorylation levels of AMPK are indicated at the bottom. **C**: Relative gene expression of *Mcp-1*, *Il-6* and *Kc* was measured by RT-PCR. **D**: Concentrations of IL-6, MCP-1 and KC in the conditioned medium of C2C12 myotubes measured by ELISA. Error bars represent SD values of biological triplicates. * = p < 0.05; ** = p < 0.01; *** = p < 0.001 compared to CM without EPS from the same experimental setup. # = p < 0.05; # # = p < 0.01 comparing fold change (FC) between the two experimental setups.

As cell-independent EPS-induced changes, as observed in hepatocytes (Fig. 4), can potentially also affect the C2C12 myotubes themselves, we treated C2C12 myotubes with non-cell EPS-CM and tested the effects on the phosphorylation of AMPK and on the mRNA expression and secretion of the myokines IL-6, MCP*-*1 and KC (Fig. 5). AMPK phosphorylation is specific for EPS stimulated contracting C2C12 myotubes, as no induction was detected when C2C12 myotubes were treated with non-cell EPS-CM (Fig. 5B). On the mRNA level, the induction of *Mcp-1* and *Kc* was largely dependent on the EPS media being conditioned by the contraction of C2C12 myotubes (Fig. 5C); a similar trend was observed for *Il-6*, but this effect failed to reach significance due to the modest inductions (Fig. 5C). To investigate whether these results could also be translated into myokine secretion into the cell culture medium, we analyzed the concentrations of IL-6, MCP-1 and KC in the media of the two different experimental settings. In agreement with the mRNA data (Fig. 5C), MCP-1 and KC, secretion was clearly induced by EPS-induced contraction, with only mild inductions observed upon treatment of C2C12 myotubes with non-cell EPS-CM (MCP-1: fold change 29.1 vs 1.6; KC: fold change 11.3 vs 1.8; Fig. 5D). The relatively mild induction of Il-6 secretion was also largely due to EPS-induced contraction (Fold change 3.7 vs 1.2; Fig. 5D). Taken together, these findings indicate that EPS can induce cell-dependent changes in C2C12 myotubes (AMPK phosphorylation, myokine secretion), but can also generate non-cell-mediated changes in cell culture media, which can affect gene expression in cells subjected to EPS-CM (e.g. *Mt1/2* and *Slc30a2* in hepatocytes). While EPS clearly represents a valuable tool in exercise research, care should be taken in experimental design to control for non-cell-mediated effects.

## Discussion

Regular exercise is very effective in the prevention and treatment of diseases^3^,^4^,^5^, but the means by which exercise mediates beneficial effects on distant target organs (e.g. liver) are largely undefined. As a model system for muscle-liver communication, we induced C2C12 myotube contraction by EPS to investigate the effects of the contraction-induced muscle secretome on hepatic gene expression. EPS specifically induced AMPK phosphorylation and myokine secretion, indicating that EPS represents a valuable tool in exercise research. When subjecting FAO hepatocytes and primary hepatocytes to CM of contracting C2C12 myotubes, expression of *Mt1*, *Mt2* and *Slc30a2* clearly increased. Fractionation of EPS-CM suggested that the main factor responsible for this phenomenon was a polar small molecule. However, a similar induction of *Mt1/2* and *Slc30a2* in FAO hepatocytes was observed when EPS-CM was generated in the absence of C2C12 myotubes. This finding suggests that EPS (and/or the electrodes) can also cause cell-independent changes in cell culture media that can subsequently affect hepatic gene expression. The identity of the molecule(s) in non-cell EPS-CM responsible for this induction of *Mt1/2* in hepatocytes remains to be established. Potential candidates include metal ions (Zn, Cu), as these can induce expression of *Mt1/2* as well as the zinc transporter *Slc30A2* that we found to be upregulated by non-cell EPS-CM too^37^,^38^. It is unlikely that these metal ions are generated by the electrodes itself, because the C-dish we used to apply the EPS to the C2C12 myotubes contains electrodes made of carbon. However, the electric pulses generated result in a redox reaction, which will result in (usually small) changes in, in our case, cell culture medium. In conclusion, our findings indicate that caution is warranted when employing EPS and that care should be taken in experimental design to control for non-cell-mediated effects, especially when cells are subjected to EPS-CM (e.g. hepatocytes in the current study).

Next to cell-independent changes, we also discovered cell-dependent effects of EPS-CM treatment. Only the CM of contracting C2C12 myotubes was able to reduce the expression of *Cyp2a3* in FAO hepatocytes. It should be noted however that this effect was not observed in primary hepatocytes and that the *Cyp2a3* gene is usually not very highly expressed in rat liver^39^. The biological relevance of the down-regulation of *Cyp2a3* in FAO hepatocytes by EPS-CM therefore remains to be established.

While our initial identification of *Mt1/2* as hepatic genes potentially regulated by the muscle secretome *in vitro* should be treated with caution (see above), both genes were also induced in mice that underwent a 12-week swimming exercise intervention^40^. Furthermore, increased hepatic *Mt* levels were shown during the recovery stage after intense acute exercise^41^. In agreement with these findings, we observed increased hepatic *Mt1* and *Mt2* expression upon a 3-week treadmill exercise intervention *in vivo* (unpublished observations). Exercise-induced expression of hepatic *Mt* genes may contribute to improved health through multiple mechanisms. The small cysteine-rich MT proteins bind metals (zinc, copper) and act as antioxidants. The ability of MT to scavenge a wide range of free radicals seems important to prevent diabetic complications, as oxidative stress is the critical initiator for diabetic onset and complications. In addition, various clinical trials have described a positive role for antioxidants in the prevention of both diabetic onset and complications^42^. MT also functions as a regulator of Zn homeostasis. A poor zinc status is very common in patients with type 2 diabetes and many physiological roles of Zn in insulin function have been indicated^43^,^44^. In addition, studies evaluating the effects of oral Zinc supplementation in patients with diabetes mellitus have demonstrated an improvement in glycaemic control and lipid parameters after zinc supplementation^45^. Several studies have indeed demonstrated a strong correlation between MT and prevention of diabetes through its antioxidant action, Zn regulation, or both^46^,^47^,^48^,^49^. Interestingly, MT increase or additional transgenic overexpression has been shown to prevent diabetic complications and liver fibrosis^43^,^50^,^51^. Furthermore, MT levels have been suggested to mediate the beneficial effect of zinc supplementation in the liver^47^. Wang *et al.* proposed a mechanism by which MT preserves GSK-3β inactivation to maintain glucose and lipid metabolism balance under diabetic conditions in cardiomyocytes^52^. If MT also inhibits GSK-3β in the liver this would result in an increased glycogen synthase activity. Previous research has indicated that both the inhibition of GSK-3β and the increase of glycogen synthase activity in the liver are beneficial for type 2 diabetic patients, which suggests that the inhibition of GSK-3β in the liver could be a third MT function beneficial for patients suffering from type 2 diabetes^53^,^54^. Taken together, these findings suggest that the exercise-induced expression of hepatic *Mt* genes may help to prevent or reverse diabetic complications.

## Additional Information

**How to cite this article**: Evers-van Gogh, I.J.A. *et al.* Electric Pulse Stimulation of Myotubes as an *In Vitro* Exercise Model: Cell-Mediated and Non-Cell-Mediated Effects. *Sci. Rep.* **5**, 10944; doi: 10.1038/srep10944 (2015).
